# Paradoxical approach towards coexistence of hepatic cyst and liver sarcoma: A case report

**DOI:** 10.1016/j.ijscr.2019.09.018

**Published:** 2019-09-24

**Authors:** Kamleshsingh Shadhu, Dadhija Ramlagun, Jordee Selvamanee Veeramootoo, Jianjie Qin, Yongxiang Xia

**Affiliations:** a Hepatobiliary Centre, The First Affiliated Hospital of Nanjing Medical University, Guangzhou Road, 300, Gulou District, Nanjing, Jiangsu, 210029, PR China; b Medical Council of Mauritius, One Way Floreal Road, Cite Magalkhan, Floreal, Mauritius

**Keywords:** Liver sarcoma, Hepatic cyst, Right upper quadrant pain, Surgery

## Abstract

**Introduction:**

Liver sarcoma is exceedingly rare and is a clinical challenge.

**Case report:**

A 69-year-old female presented with right upper quadrant pain after a fall. Her CT scan showed multiple cysts. She was initially diagnosed as hepatic cysts with hemorrhage and underwent puncture and drainage of liver cysts. But the cysts relapsed quickly. Then she underwent fenestration of the hepatic cysts. The liver cysts reoccurred again accompanied by massive bleeding and obstructive jaundice. She underwent right trihepatectomy and her final pathological report showed liver sarcoma. However, the hepatic sarcoma reoccurred again. Its’ diagnosis and treatment process are extremely complex. In the end, she had a bad ending.

**Conclusion:**

We believe that timely diagnosis and complete excision of liver sarcoma are very important.

## Highlights

• Coexistence of hepatic cyst and liver sarcoma.
• Recurrence of hepatic cysts.
• Surgical approach has been mentioned as the treatment modality.
• The roles of chemotherapy and radiotherapy are still controversial.

## 1 Introduction

Liver sarcoma is exceedingly rare and has been reported by literatures [1,2]. We report a case of a 69-year-old female whereby diagnosis and treatment were a clinical challenge for our team. This case report has been reported in line with the SCARE criteria [3].

## 2 Case presentation

**Fig. 1:**
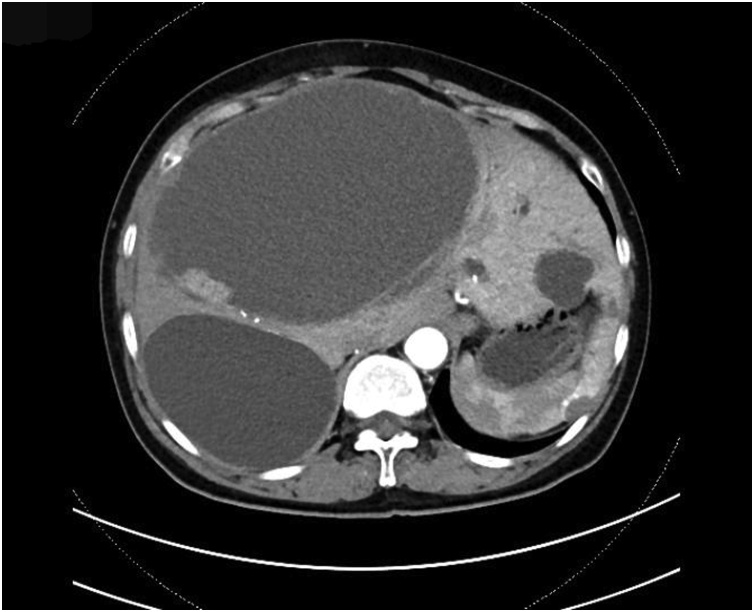
CT scan showing hepatic multiple cysts.

**Fig. 2:**
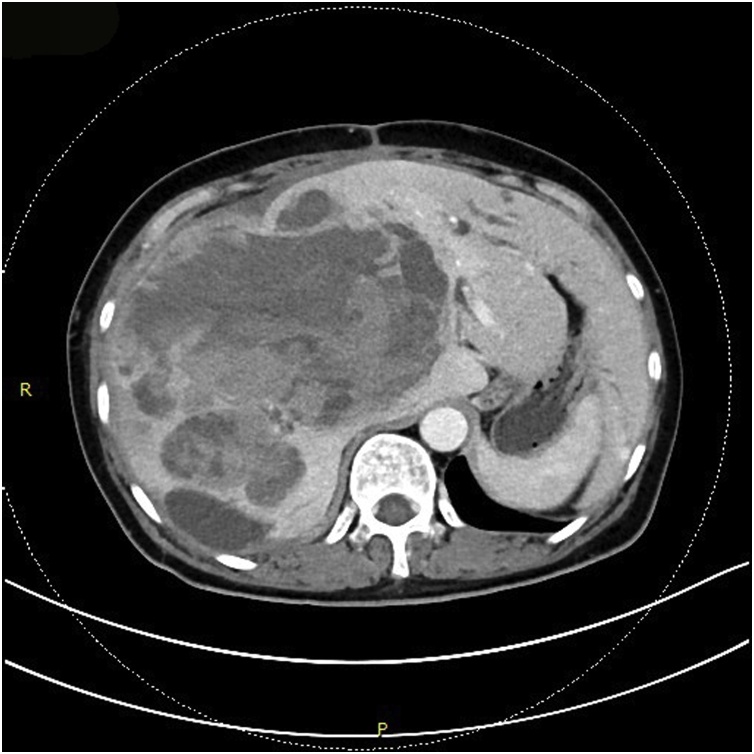
CT scan showing recurrence of hepatic multiple cysts.

**Fig. 3:**
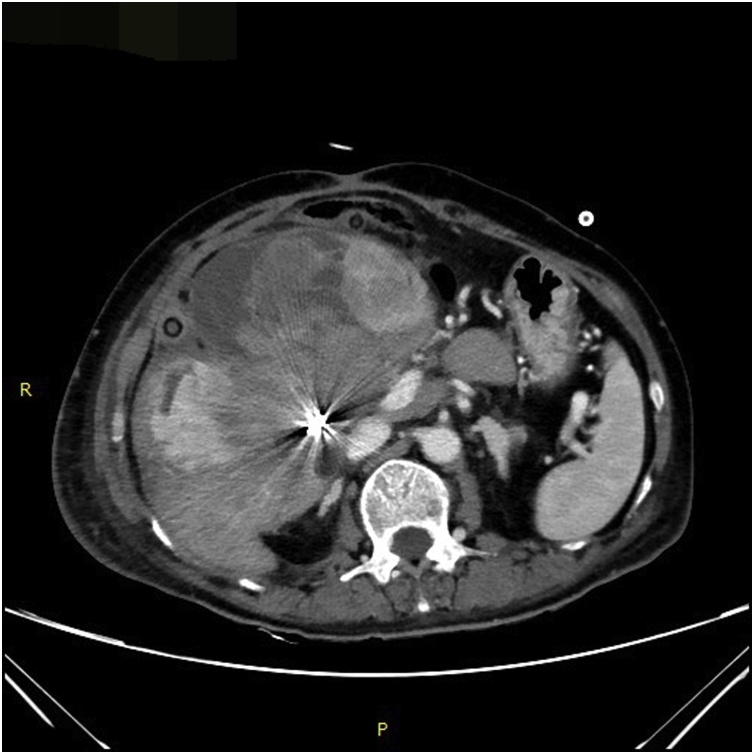
CT scan showing atrophy of liver, dilatation of bile duct, compression and displacement of hepatic portal system.

**Fig. 4:**
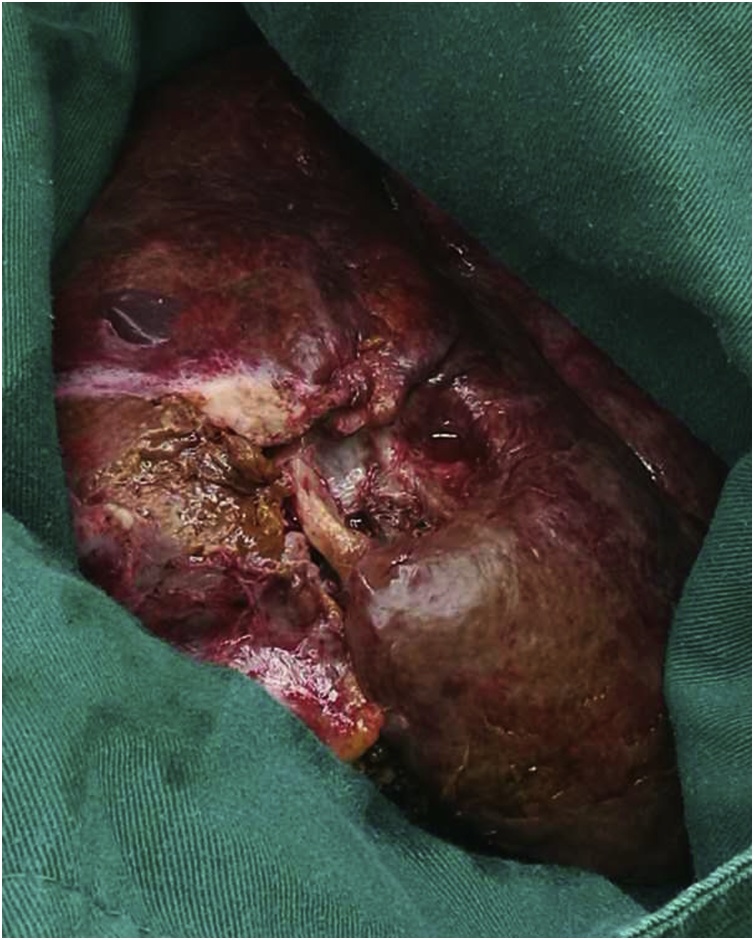
The resected sample.

**Fig. 5:**
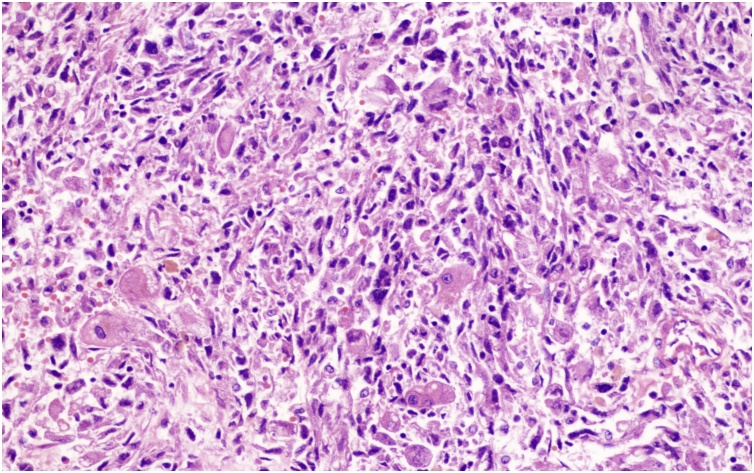
HE stain of resected sample (magnifying power ×100).

A 69-year-old female patient present to our hospital with right upper abdominal severe pain for several hours after a fall. It was accompanied by fatigue. Her upper abdominal CT scan showed intrahepatic round cysts with the largest of size 14 cm × 16 cm. Irregular high-density shadows were seen on the edges of the cysts (Fig. 1). She was initially diagnosed as hepatic cysts with hemorrhage and underwent puncture and drainage of liver cysts. About 600 mL of dark red liquid was drained from the largest of the liver cysts, which improved the symptoms, but the cysts relapsed quickly. She underwent partial hepatectomy (fenestration of hepatic cysts), cholecystectomy and T-tube drain placement in the common bile duct. During the surgery, we found a large cyst large cyst of 20 cm diameter occupying the right lobe of the liver. The common bile duct was dilated about 1.5 cm. We resected the thinner part of the cyst wall and about 2000 mL of bile-like liquid mixed with blood clots and fresh blood were drained. Blood clots were mostly concentrated at the thicker part of the cystic wall and we biopsied the thicker cyst wall. Postoperative pathological report was simple hepatic cyst. 10 days post-operatively, the patient was complaining about paroxysmal abdominal pain which was also colicky. She had flatus but no defecation. Conservative treatment was in vain and hence, on POD 11 she underwent laparotomy as intestinal obstruction was suspected. It was observed that the transverse colon was wrapped around the T tube in the abdominal cavity. There were adhesive tissues due to which the intestine and transverse colon were folded. The adhesive tissues were relieved. 17 days after the surgery, the patient was discharged. 68 days later, the patient came back complaining about right upper abdominal pain and jaundice. Conservative management had no effect. Her CT scan shows a mixture of density at the liver which may be hemorrhage, recurrence of multiple hepatic cysts (Fig. 2). The intrahepatic bile duct was dilatated. The huge cyst compressed the portal system of the liver. There was obstructive jaundice, the bilirubin was 282 μmol/L. There was also intracystic hemorrhage, her blood hemoglobin was 71 g/L. 3 days after admission, she underwent laparotomy whereby there was clearance of hepatic cysts lesions and homeostasis with gauze filling and compression. During the surgery, the giant cyst of the right trilobe had a diameter of 18 cm. The cystic wall was tightly adhered to the diaphragm and intestine. About 3000 mL of large amount of blood clots, necrotic tissue and blood were lost and approximately 2000 mL of fresh blood were lost. The heart beat was 150 beats per minute and the blood pressure about 70/40 mmHg. The patient was therefore transferred to the ICU on mechanical ventilation. 3 days later, right hepatic artery embolization was performed, then laparotomy was done to remove the homeostatic gauzes. 12 days after the surgery, her CT scan showed intracystic, hematoma and right hepatic artery embolization (Fig. 3). 22 days later, in order to relieve completely the repeated episodes of hepatic cyst hemorrhage, right trihepatectomy was carried out (Fig. 4). The procedure was hard and about 8000 mL of blood was lost so the patient was transferred to ICU. 7 days afterwards she was transferred to our department and her pathological report showed malignant tumor, cystic lesion of 6 cm × 5 cm × 4 cm, solid area of the lesion is 3 cm × 2 cm × 2 cm, the final pathological report showed liver sarcoma (Fig. 5). Although it was R0 resection, liver sarcoma relapsed 2 months later.

## 3 Discussion

Although liver sarcoma was suggested to have a slight male predominance by WHO classification, Chan et al. found that most of cases involved female patients, like in our case. Our patient presented with right upper quadrant pain which was also reported by Yi et al. [2]. CT scan as the gold standard as imaging modality has also been favored in literatures [2]. Our case is mainly hepatic cyst with partial parenchymal lesions which was misdiagnosed as hematoma. Surgical approach has been mentioned as the treatment modality, and the roles of chemotherapy and radiotherapy are still controversial. Complete resection of tumors is very important, positive surgical margins are associated with a higher risk of local recurrence, distant metastasis and death [4]. In our case, puncture and partial resection may lead to rupture of tumors causing the spread of tumors. Some authors [5,6] have reported successful outcome of patients with postoperative chemotherapy and radiotherapy. Improved survival rates, ranging from 70% to 100%, have been reported with multimodal therapy.

We believe that timely diagnosis and complete excision of liver sarcoma are very important. Our case is a hepatic sarcoma coexisting with giant cysts and intracystic hemorrhage of the liver after trauma, so it leaded to missed diagnosis.

## Sources of funding

No Funding received.

## Ethical approval

The Ethics committee of First Affiliated Hospital of Nanjing Medical University approved this case report.

## Consent

Written informed consent has been obtained from the patient and will be provided to the editor upon request.

## Author’s contribution

All authors should have made substantial contributions to all of the following: (1) the conception and design of the study, or acquisition of data, or analysis and interpretation of data, (2) drafting the article or revising it critically for important intellectual content, (3) final approval of the version to be submitted.

## Registration of research studies

Not applicable.

## Guarantor

Jianjie Qin.

## Provenance and peer review

Not commissioned, externally peer-reviewed.

## Declaration of Competing Interest

No Conflict of Interest by the authors.
